# Generation and characterization of NGLY1 patient-derived midbrain organoids

**DOI:** 10.3389/fcell.2023.1039182

**Published:** 2023-02-16

**Authors:** Joshua Abbott, Mitali Tambe, Ivan Pavlinov, Atena Farkhondeh, Ha Nam Nguyen, Miao Xu, Manisha Pradhan, Tate York, Matthew Might, Karsten Baumgärtel, Steven Rodems, Wei Zheng

**Affiliations:** ^1^ National Center for Advancing Translational Sciences, National Institutes of Health, Bethesda, MD, United States; ^2^ Institute for Cell Engineering, Johns Hopkins University School of Medicine, Baltimore, MD, United States; ^3^ 3Dnamics, Inc., Baltimore, MD, United States; ^4^ NeuroScience Associates Inc, Knoxville, TN, United States; ^5^ University of Alabama at Birmingham, Birmingham, AL, United States; ^6^ Travere Therapeutics, San Diego, CA, United States

**Keywords:** NGLY1, midbrain organoids, dopaminergic neurons, GABA, GFAP

## Abstract

NGLY1 deficiency is an ultra-rare, autosomal recessive genetic disease caused by mutations in the *NGLY1* gene encoding N-glycanase one that removes N-linked glycan. Patients with pathogenic mutations in NGLY1 have complex clinical symptoms including global developmental delay, motor disorder and liver dysfunction. To better understand the disease pathogenesis and the neurological symptoms of the NGLY1 deficiency we generated and characterized midbrain organoids using patient-derived iPSCs from two patients with distinct disease-causing mutations–one homozygous for p. Q208X, the other compound heterozygous for p. L318P and p. R390P and CRISPR generated NGLY1 knockout iPSCs. We demonstrate that NGLY1 deficient midbrain organoids show altered neuronal development compared to one wild type (WT) organoid. Both neuronal (TUJ1) and astrocytic glial fibrillary acid protein markers were reduced in NGLY1 patient-derived midbrain organoids along with neurotransmitter GABA. Interestingly, staining for dopaminergic neuronal marker, tyrosine hydroxylase, revealed a significant reduction in patient iPSC derived organoids. These results provide a relevant NGLY1 disease model to investigate disease mechanisms and evaluate therapeutics for treatments of NGLY1 deficiency.

## Introduction

*NGLY1* is an evolutionarily conserved gene that has a critical role in the endoplasmic reticulum associated degradation (ERAD) pathway. *NGLY1* deficiency is a ultrarare genetic disorder that is associated with an array of symptoms that include seizures, hyperkinetic movement, alacrima, developmental delay, and abnormal liver function (Caglayan et al., 2015; Lam et al., 2017). *NGLY1* deficiency represents the first known congenital disease of deglycoslyation (CDDG) with the first diagnosis of NGLY1 deficiency in 2012. It is considered an ultrarare genetic disorder with approximately 75 patients diagnosed as of 2022 (NORD). Pathogenic variants of *NGLY1* are caused by biallelic mutations and follow an autosomal recessive pattern of inheritance (Lam et al., 2017). Nearly all known *NGLY1* mutations result in complete loss of NGLY1 protein with reduced mRNA levels.

The best characterized function of NGLY1 is the removal of N-glycans from misfolded glycoproteins that are subsequently directed for proteasomal degradation as a part of the ERAD cellular process (Suzuki et al., 2003; Suzuki, 2015; Suzuki et al., 2015; Suzuki et al., 2016). Despite multiple studies performed in model organisms (Tambe et al., 2019; Han et al., 2020; Talsness et al., 2020), the pathogenesis of NGLY1 deficiency is still poorly understood. Recent studies focusing on neurological phenotypes of NGLY1 disorder have shown disruptions in ion channels, monoamine pathways and dysregulation in stress responses (Owings et al., 2018; Mueller et al., 2020; Hope et al., 2022).

Since the midbrain area of the brain controls the motor system and is implicated in movement disorders like Parkinson’s disease, we reasoned that a midbrain organoid model would help us understand how NGLY1 deficiency may cause a movement disorder. Hence, we generated midbrain organoids from iPSCs derived from patients with different NGYL1 mutations: p. Q208X (NGLY1 519) and p. L318P/R390P (NGLY1 594) (Li et al., 2019). In parallel, we generated midbrain organoids from a pair of wild type and CRISPR knockout (KO) NGLY1 iPSCs to minimize the effects of genetic background in individual patients. Our data describes alterations in key brain markers in the development of NGLY1 deficient midbrain organoids. Specifically, we observe that midbrain organoids containing NGLY1 patient mutations exhibit a drastic reduction in tyrosine hydroxylase, indicating possible disruption in the dopaminergic pathway. Furthermore, we identified altered pathways associated with NGLY1 deficiency in midbrain organoids through gene set enrichment analysis of RNA-seq and global-proteomic profiling. Development of a midbrain organoid model for NGLY1 deficiency enables a better understanding of the disease pathogenesis and may also serve as a potential screening platform to develop therapeutics for NGLY1 deficiency.

## Materials and methods

### IPS cell line generation and culture

Patient skin fibroblasts were obtained from Coriell Cell Repositories (GM25344, GM22602), and cultured in DMEM supplemented with 10% fetal bovine serum, 100 units/mL penicillin and 100 μg/mL streptomycin in a humidified incubator with 5% CO2 at 37°C. To generate the iPS cells, the non-integrating CytoTune-Sendai viral vector kit (A16517, Thermo Fisher Scientific) containing OCT3/4, KLF4, SOX2 and C-MYC pluripotency transcription factors was employed to transduce the patient fibroblasts. Human iPSCs were cultured in mTeSR™1 (STEMCELL Technologies) on Matrigel (Corning, 354,277)-coated plates at 37°C in humidified air with 5% CO2 and 5% O2.

### Midbrain generation from iPSC

The method for generating midbrain organoids from iPSCs was adapted from a previously published literature (Qian et al., 2018). Briefly iPSCs (Corriel) were dissociated into single cells with Versene solution (Gibco) and plated into a well of an Aggrewell plate (Stemcell Technologies Inc.) at 3,000 cells per microwell. The plate was spun down according to manufacturer’s recommendation in StemFlex medium (Gibco) supplemented with 10 µM ROCK inhibitor Y-27632 (Tocris 1254). After overnight incubation, about 300 spheroids were collected and transferred into a well of a 6-well ultralow attachment plate (Corning) containing midbrain differentiation medium one consisting of DMEM/F12 (Gibco), 20% Gibco KnockOut Serum Replacement (Gibco), 1X Glutamax (Gibco), 1X MEM Non-essential Amino Acid solution (Gibco), 55 µM 2-Mercaptoethanol (Gibco 21985023), 1X Pen/Strep (Gibco), 100 nM LDN-193189 (Tocris 6053), 10 µM SB-431542 (Tocris 1614), 2 µM Purmorphamine (Tocris 4551), 200 ng/mL Sonic hedgehog (Peprotech 100–45), and 200 ng/mL FGF-8b (Peprotech 100–25). After 5 days of culture, the medium was replaced with midbrain differentiation medium two consisting of DMEM/F12, 1X N-2 Supplement (Gibco), 1X Glutamax, 1X MEM Non-essential Amino Acid solution, 1X Pen/Strep, 100 nM LDN-193189, 3 µM CHIR-99021 (Tocris 4953), 2 µM Purmorphamine, 200 ng/mL Sonic hedgehog, and 200 ng/mL FGF-8b. At this stage and thereafter, the plate was placed on an orbital shaker rotating at 120 RPM in an incubator. On day 7, midbrain organoid differentiation medium three consisting of DMEM/F12, 1X N-2 Supplement, 1X Glutamax, 1X MEM Non-essential Amino Acid, 1X Pen/Strep, 100 nM LDN-193189, 3 µM CHIR-99021 was added. After seven additional days of incubation, the medium was replaced with final midbrain organoid differentiation medium consisting of Neurobasal medium (Gibco), 1X B-27 Supplement (Gibco), 1X Glutamax, 55 µM 2-Mercaptoethanol, 1X Pen/Strep, 0.2 mM Ascorbic acid (Sigma Aldrich A92902), 0.5 mM cAMP (Acros Organics), 20 ng/mL Brain-derived neurotrophic factor (Peprotech 450–02), 20 ng/mL Glial-derived neurotrophic factor (Peprotech 450–10), 1 ng/mL Transforming growth factor beta (Peprotech 100–21).

### Generation of CRISPR NGLY1 knockout iPSC

​​The U6-gRNA-CAG promoter of pCAG-SpCas9-GFP-U6-gRNA (Addgene #79144) was replaced at PciI-NcoI with a T7 promoter plus Kozak sequence. The EcoRV-FseI fragment was subsequently replaced with the EcoRV-FseI fragment containing the three point mutations of high-fidelity eSpCas9 from Feng Zhang lab at MIT (Addgene # 71814). Following linearization with NotI, mRNA was transcribed using NEB #E2060S HiScribe T7 ARCA mRNA Kit (with tailing) plus 5mCTP and PseudoUTP from TriLink Biotechnologies (N-1014 and N-1019). The transcription reaction was purified with NEB Monarch Total RNA Miniprep Kit (T2010G) and quantified with a Nanodrop 2000c. 30 min before transfection, HT268A iPSCs were dissociated with TrypLE, counted, and plated at 6 × 10^5 cells per well of a Matrigel coated 6-well plate in 2.5 mL E8 + 1X RevitaCell supplement. Transfection was done using 4 µL Lipofectamine Stem Reagent in 200 µL OptiMEM with 20 pmol synthetic gRNA from Life Technologies (A35511) to target NGLY1 exon 2 (ACT​AGA​CTC​TTG​CCT​GTC​AG) and 1800 ng eSpCas9-GFP mRNA. On the second day post transfection, GFP positive iPSCs were sorted onto Matrigel coated 96-well plates containing 100 µL E8-Flex plus 1X CloneR™ supplement (Stemcell Technologies #05889) at 3 cells per well. After 72 h 100 µL of E8 Flex was added per well. Subsequent medium changes were every other day at 50% of the volume with E8-Flex. Wells with surviving clones after 10 days were expanded to isolate gDNA for screening. A genomic fragment spanning the gRNA target site was amplified with forward primer (5′- CCA​TTG​GTT​TAG​GGA​AGA​AAG​AAA), reverse primer (5′- CCT​TGG​GTT​GCA​TGT​ATG​ACC), and Phusion^®^ Hot Start Flex DNA Polymerase (NEB M0535) and Sanger sequenced (Eurofins Genomics) using primer 5′- CCT​AGG​TCA​TGT​TTG​TTG​AAA​GTA​A to identify KO clones. Clones with homozygous frameshift mutations, such as ACT​AGA​CTC​TTG​CCT​GT**T**CAG with 1bp **T** insertion, are likely homozygous KO clones.

### Embedding, sectioning and IHC

At least five midbrain organoids were pooled from three separate batches for each time point and treated overnight with 20% glycerol and 2% dimethylsulfoxide to prevent freeze-artifacts. The specimens were then embedded in a gelatin matrix using MultiBrain^®^/MultiCord^®^ Technology (NeuroScience Associates, Knoxville, TN).

The blocks were rapidly frozen, after curing by immersion in 2-Methylbutane chilled with crushed dry ice and mounted on a freezing stage of an AO 860 sliding microtome. The MultiBrain^®^/MultiCord^®^ blocks were sectioned in coronally with desired micrometer *μ*) setting on the microtome. All sections were cut through the entire length of the specimen segment and collected sequentially into series of 24 containers. All containers contained Antigen Preserve solution (50% PBS pH7.0, 50% Ethylene Glycol, 1% Polyvinyl Pyrrolidone); no sections were discarded.

For IHC, free floating sections were stained with desired stain. All incubation solutions from the primary antibody onward use Tris buffered saline (TBS) with Triton X-100 as the vehicle; all rinses are with TBS.

The sections were immunostained with the primary antibodies (Table I) overnight at room temperature. Vehicle solutions contained Triton X-100 for permeabilization. After washing, a fluoro-tagged or biotinylated secondary antibody was applied. Sections stained with a biotinylated secondary antibody (Table I) were treated with a fluorescent tagged streptavidin and washed. All sections were then mounted on gelatin coated glass slides and air dried. The slides were dehydrated in alcohols, cleared in xylene and coverslipped.

### Transmission electron microscopy

At least five 90-day midbrain organoids were pooled from three separate batches and were fixed with 2% glutaraldehyde and 4% formaldehyde in 0.1 M Sodium Cacodylate buffer for an hour at room temperature. Each organoid was transferred to a glass vial containing 0.1 M Sodium Cacodylate buffer and organoids were washed with 0.1 M Sodium Cacodylate buffer two times for 10 min. After the second buffer wash, the organoids were post-fixed with 1% osmium tetroxide for an hour in the dark. After an hour post fixation, organoids were again washed with 0.1 M Sodium Cacodylate buffer twice for 10 min Midbrain organoids were washed once with 0.1 N Sodium Acetate buffer and then stained with 0.5% Uranyl Acetate for an hour in the dark. After *en block* staining, organoids were washed with 0.1 N Sodium Acetate buffer twice for 10 min. Organoids were then subjected to gradual dehydration in the order of 35%, 50%, 70% and 95% ethanol twice for each step for 10 min and three times with 100% ethanol for 10 min. After the last step of 100% ethanol rinse, organoids were further dehydrated in Propylene oxide (PO) for 10 min three times. After the last step in PO, organoids were infiltrated in 50:50 epoxy resin and PO overnight at room temperature.

The next day, after overnight infiltration, each organoid was removed from 50:50 epoxy resin and PO, blotted, and embedded in a plastic mold containing 100% pure epoxy resin and transferred to a 55°C oven for 48 h. Epoxy resin (PolyScience Resin) ingredients consisted of a mixture of Poly/Bed 812 embedding Media, Dodecenylsuccinic Anhydride (DDSA), Nadic Methyl Anhydride (NSA) and DMP-30 to solidify the resin. After 48 h, organoids were taken out of the oven, Each Organoid embedded in resin mold was ultra-thin sectioned with a UC6 Leica Microtome at 70 nm. The ultra-thin sections were picked up on a 150 Copper mesh grid and were examined under a Hitachi H7600 transmission electron microscope. The grids were post-stained with 1:1 0.5% Uranyl Acetate in ddH2O and 70% ethanol for 2 min and then rinsed with ddH2O for four times. Then organoids stained with 1:1 Lead Citrate and ddH2O for 2 min and rinsed with ddH2O four times. The grids were carbon coated with a TedPella/Cressington Evaporator and imaged in a Hitachi H7600 transmission electron microscope at 80 KeV.

### Scanning electron microscopy

90-day midbrain organoids were fixed and dehydrated as described above. After the last step of 100% ethanol rinse, organoids were air-dried with tetramethylsilane (TMS) three times under the hood for 10 min. After air-drying, each organoid was carefully picked up by a tweezer and attached to a carbon tape on a SEM stub and then transferred to an Edwards vacuum system and coated with highly conductive Au/Pd metal uniformly by a rotary tilting stage for 3 min. After Au/Pd coating, each organoid was transferred to a Hitachi S4500 scanning electron microscope for higher resolution imaging at 5.0 KeV.

### Western blotting analysis

At least five midbrain organoids from three different batches, at day 60 and day 90 timepoints, were lysed in Western ready-rapid protein extraction buffer (BioLegend), boiled and protein concentrations were determined using Pierce BCA protein assay (Thermo Scientific). Equal amount of denatured proteins were separated *via* SDS–polyacrylamide gel electrophoresis on a 4%–20% Mini-PROTEAN TGX precast protein gel (BioRad) and transferred to a polyvinylidene difluoride (PVDF) membrane (pore size, 0.45 mm). The membrane was blocked in Pierce Protein-Free blocking buffer (ThermoFisher Scientific) and probed overnight with an appropriate primary antibody (Table 1). The membrane was treated with a secondary anti-mouse or anti-rabbit, horseradish peroxidase-conjugated antibody (1:10,000). Western blots were developed using Pierce ECL Western Blotting Substrate (Thermo Scientific) and chemiluminescence images were obtained using G:Box Chemi XRQ imaging system (Syngene). Quantitative analysis of the western blotting data was carried out on three different blots using ImageJ software.

### RNA extraction and RNA-seq

Total RNA isolation from at least three 90-day midbrain organoids from three separate batches was performed using RNeasy Midi kit (cat #75144, Qiagen, Gaithersburg, MD). RNA quality was determined using a Bioanalyzer 6100 (Agilent, Santa Clara, CA). Directional RNA-seq was performed with 100 ng of total RNA using the TruSeq Stranded mRNA Library Prep Kit (Illumina, San Diego, CA), and 125 bp end sequence readers were generated using the HiSeq 2,500 platform (Illumina, San Diego, CA).

### Protein extraction and TMT-10plex MS

Total protein extraction from at least three 90-day midbrain organoids from three separate batches were prepared following manufacturer protocol (Poochon Scientific, Gaithersburg, MD). The protein concentration of the supernatants was determined by a BCA™ Reducing Reagent compatible assay kit (Thermo Scientific; Rockford, IL). 100 μg of protein lysate from each sample was used for in-solution trypsin digestion. Pre-digestion along with digested samples were examined by SDS-PAGE Coomassie stain to check digestion efficiency. Isobaric labeling was carried out using the TMT-10plex kit (#90110, ThermoFischer Scientific, San Jose, CA) according to the product manual.

Labeled peptides were resuspended in 10 mM TEABC. To determine the labeling efficiency before fractionation by analysis of a small aliquot of the sample (1%). A minimum 95% of labeling efficiency is required. The fractionation of TMT-10plex labeled peptide mixture is carried out using an Agilent AdvanceBio Column (2.7 μm, 2.1 × 250 mm) at Solvent A (10 mM TEABC, pH 8.0) and an Agilent UHPLC 1290 system. The separation is performed by running a gradient of Solvent B (10 mM TEABC, pH 8.0, 90% ACN) and Solvent A (10 mM TEABC, pH 8.0) at the flow rate 250 μL/min. The elute fractions are collected into a 96-well plate using a 1260 series auto-sample fraction collector. The 96 elute fractions are further combined into 24 fractions according to collection time.

The LC/MS/MS analysis was carried out using a Thermo Scientific Q-Exactive hybrid Quadrupole-Orbitrap Mass Spectrometer and a Thermo Dionex UltiMate 3,000 RSLCnano System. Each peptide fraction from a set of 24 fractions is loaded onto a peptide trap cartridge at a flow rate of 5 μL/min. The trapped peptides were eluted onto a reversed-phase 20 cm C18 PicoFrit column (New Objective, Woburn, MA) using a linear gradient of acetonitrile (3%–36%) in 0.1% formic acid. The elution duration was 110 min at a flow rate of 0.3 μL/min. Eluted peptides from the PicoFrit column is ionized and sprayed into the mass spectrometer, using a Nanospray Flex Ion Source ES071 (ThermoFischer Scientific, San Jose, CA) under the following settings: spray voltage, 1.8 kV, Capillary temperature, 250°C. 24 fractions were analyzed sequentially. The Q Exactive instrument was operated in the data dependent mode to automatically switch between full scan MS and MS/MS acquisition. Survey full scan MS spectra (m/z 300–1800) was acquired in the Orbitrap with 70,000 resolutions (m/z 200) after accumulation of ions to a 1 × 106 target value based on predictive automatic gain control (AGC). Dynamic exclusion was set to 20 s. The 15 most intense multiply charged ions (z ≥ 2) were sequentially isolated and fragmented in the octopole collision cell by higher-energy collisional dissociation (HCD) using normalized HCD collision energy 28% with an AGC target 1 × 105 and a maxima injection time of 100 m at 17,500 resolutions.

### IHC image analysis and quantification

Image analyses were performed using the HALO software from IndicaLabs. Area Quantification module was used to quantify the positively stained region for each slide. Analysis parameters (signal intensity ranges for low, moderate, and weak intensities) were set and applied to all images for a given stain. The finalized analysis algorithm was run on all images and the generated data were exported and organized in a Microsoft Excel spreadsheet. All graphs and summary data were generated from the raw data using GraphPad Prism 9.

### Statistical analysis

Statistical analysis was performed using the GraphPad Prism software. Statistical significance was performed by one-way ANOVA analysis Brown-Forsythe ANOVA, Welch’s ANOVA, and *p*-values were corrected for multiple hypothesis testing using Dunnett’s T3 multiple comparisons test. Details about the replicates and specific statistical analyses can be found in the figure legends. Statistical significance was denoted by * = (*p* ≤ 0.05), ** = (*p* ≤ 0.01), *** = (*p* ≤ 0.001), **** = (*p* ≤ 0.0001), No annotation was given to non-significant data.

### RNA-seq and GSEA analysis

Quality control of FASTQ files were evaluated using FastQC (v0.11.5), raw reads were trimmed for Illumina adapter in Trimmomatic (v0.36). Sequence alignment was performed using STAR (v2.5.2. a) with reverence assembly GRCh38. p3 and Ensembl annotation 82 with ENCODE options.

All secondary analyses were performed in the R statistical environment (R version 4.0.2). Genes were filtered to keep genes with more than one count per million (CPM) in at least 75% of the samples. PCA was performed using PCAtools (v2.4.0) on TMM normalized data using edgeR (v3.16.5). Statistical analysis to determine differentially expressed genes was performed with limma (v3.48.1). Dispersion estimation was performed using the voom function and statistical analysis was completed using eBayes. Genes were considered differentially expressed if Log2 fold change was > |1| with a Benjamini–Hochberg false-discovery rate below 5%.

Gene set enrichment analysis (GSEA) was performed using ClusterProfiler (v4.0.0) utilizing the NCATS BioPlanet for pathway and gene set determination. Pathways were determined to be of interest with a Benjamini–Hochberg false-discovery rate below 25%.

### MS data and pathway analysis

MS Raw data files were searched against the human protein sequences database obtained from UniprotKB website using the Proteome Discoverer 1.4 software (ThermoFischer Scientific, San Jose, CA) based on the SEQUEST and percolator algorithms. The false positive discovery rate (FDR) was set at 5%. The resulting Proteome Discoverer Report contains all assembled proteins with peptides sequences and peptide spectrum match counts (PSM#) and TMT-tag based quantification ratio.

TMT-tag based quantification was used for determining the relative abundance of proteins identified from each set of 10 samples using one TMT-10plex. The common reference was used for calculation of the ratio in each set of a TMT-10plex analysis. The relative abundance of proteins in each TMT-set was normalized using the reference mix.

Gene set enrichment analysis (GSEA) was performed using ClusterProfiler (v4.0.0) utilizing the NCATS BioPlanet for pathway and gene set determination. Pathways were determined to be of interest with a Benjamini–Hochberg false-discovery rate below 25%.

## Result

### Generation of midbrain-specific healthy and NGLY1 disease organoids

**FIGURE 1:**
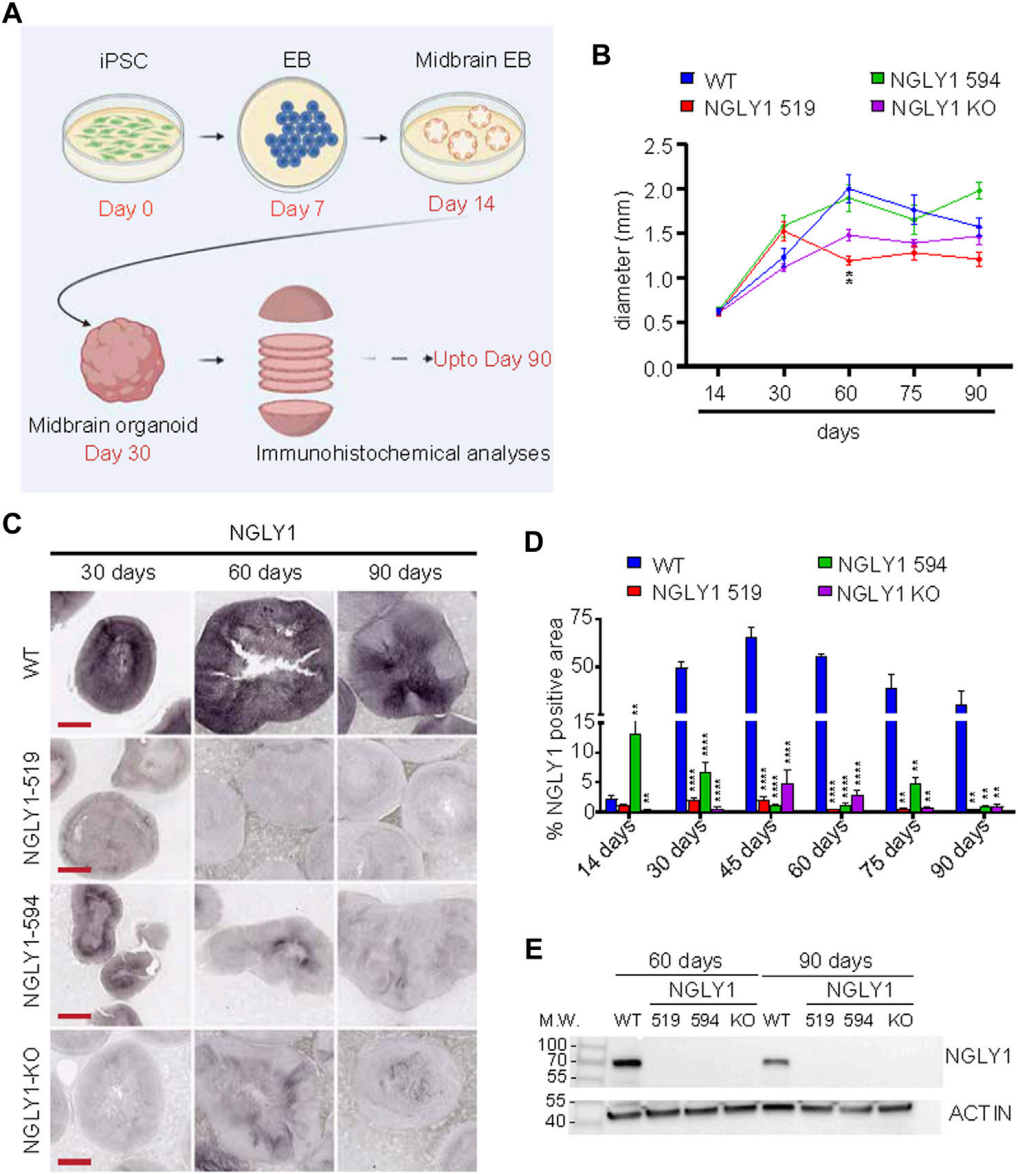
NGLY1 disease midbrain organoids do not express NGLY1 protein. **(A)** A schematic showing the different stages of midbrain organoid development. **(B)** A graph showing changes in midbrain organoid size, presented as diameter (mm) through development and maturation. **(C)** Representative images using brightfield microscopy for midbrain organoids stained with antibody against NGLY1 at different timepoints (45, 60 and 90 days). Scale bars: 500 µM. **(D)** Graph showing percent total NGLY1 positive signal in different organoids at different timepoints. **(E)** A representative western blot showing NGLY1 expression in cell lysates obtained from control and NGLY1 disease midbrain organoids. Statistical significance determined by multiple *t*-test analysis denoted by * = (*p* ≤ 0.05), ** = (*p* ≤ 0.01), *** = (*p* ≤ 0.001), **** = (*p* ≤ 0.0001).

The midbrain organoids were prepared by adapting a protocol described previously (Qian et al., 2018). The organoids were generated from wild type (WT) and NGLY1 patient derived hiPSC containing two different NGYL1 mutations: p. Q208X (NGLY1 519) and p. L318P/R390P (NGLY1 594), as well as a CRISPR generated NGLY1 KO line. Briefly, embryoid bodies (EBs) were generated from hiPSC cultures. Midbrain-specific growth factors and compounds were sequentially added at different stages of development. Next, the organoids were transferred to a spin bioreactor to allow maturation up to 90 days (Figure 1A). In order to determine the effect of NGLY1 mutations and NGLY1 KO on the overall development of the midbrain organoids, we tracked the size of the organoids through development and maturation. The WT and NGLY1 594 organoids had similar sizes and reached their biggest size at around day 60. Interestingly, the NGLY1 519 and NGLY1 KO organoids were consistently smaller than the WT organoids and showed a 30%–50% reduction in size at the 60-day time point (Figure 1B). The dramatic reduction in organoid size suggests an important role of NGLY1 in the early stages of midbrain development.

To assess NGLY1 expression through the development of midbrain organoids, we performed immunohistochemical and western blot analysis against NGLY1. WT midbrain organoids showed significant expression of NGLY1 protein starting from day 30, reaching peak around day 45 that maintained to day 90. As expected, NGLY1 519 and NGLY1 KO midbrain organoids showed no NGLY1 expression at any time point determined by both methods (Figures 1C–E). Interestingly, NGLY1 594 midbrain organoids showed slight staining for NGLY1 in the earlier stages of organoid development (day 14 and day 30) but failed to maintain NGLY1 expression in mature midbrain organoids (Figures 1C,D) that may explain the similar size of NGLY1 594 organoids at the day 60 compared to the WT organoids.

### NGLY1 deficient midbrain organoids have altered development

**FIGURE 2:**
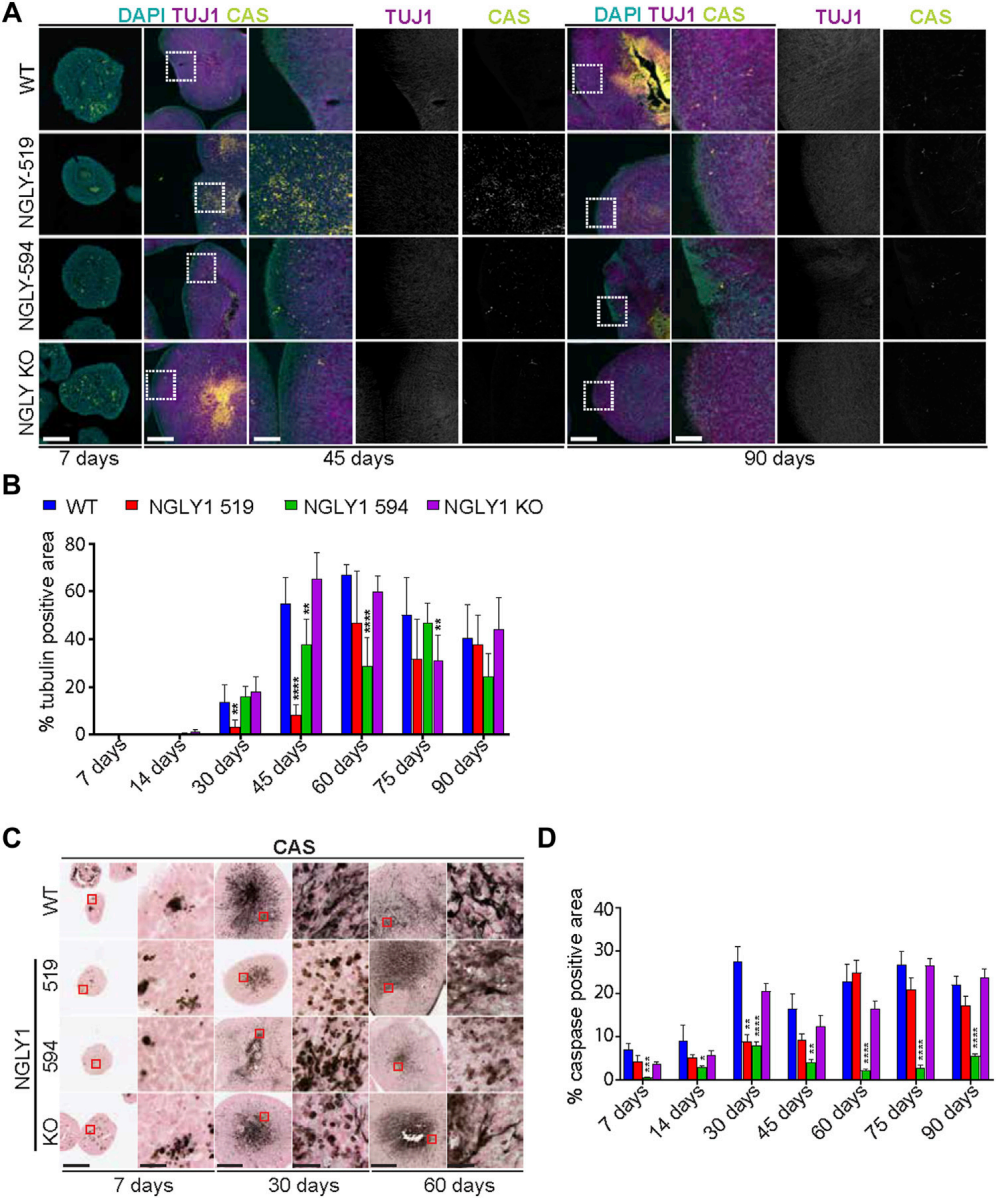
Development and health of organoids as determined by βIII Tubulin (TUJ1) and Caspase3 (CAS) staining in wild type (WT), mutant (NGLY1-519 and NGLY1-594) and knockout (NGLY1 KO) midbrain organoids. **(A)** Immunofluorescence images at ×5 and ×20 magnification showing DAPI (cyan), TUJ1 (magenta) and CAS (yellow) staining in each line. At 30 days the mutant and KO lines develop a distinct TUJ1 staining pattern relative to the WT, with significantly less density at the edge of the organoid. Scale bars: 125 μm (5x), 25 μm (20x). White dashed line boxes indicate regions of magnification. **(B)** Quantification of the total TUJ1-positive area in each line at each time point. Bar graph shows the percent of the total tissue area of each line that was positive for TUJ1. Time points where the percentage of TUJ1-positive area in WT organoids was close to zero were excluded. **(C)** Immunohistochemistry images at ×4 and ×40 magnification showing CAS staining in each organoid line. As organoids grow in size, regions of intense CAS staining develop at the center of the wild type and mutant lines. Scale bars: 250 μm (4x), 25 μm (40x). Red boxes indicate regions of magnification. **(D)** Quantification of the total CAS-positive area in each line at each time point. Bar graph shows the percent of the total tissue area of each line that was positive for CAS. Data representative of all slices derived from three midbrain organoids. Statistical significance determined by multiple *t*-test analysis denoted by * = (*p* ≤ 0.05), ** = (*p* ≤ 0.01), *** = (*p* ≤ 0.001), **** = (*p* ≤ 0.0001).

To dissect the cell populations that may contribute to reduced size, we co-stained organoid sections from each iPSC line with the antibodies against βIII Tubulin (TUJ1), a neuron-specific form of tubulin, and cleaved Caspase 3 (CAS), an apoptosis marker (Figure 2) (D'Amelio et al., 2010; Monzel et al., 2017). After 2 weeks of differentiation, all organoid lines started to express a small amount of TUJ1 signifying the maturation of the EB into MOs (Figure 2B). However, as development progressed, differences between the WT and the other lines began to emerge and are especially apparent at days 45 and 60 (Figure 2B). At 45 days the overall quantity of TUJ1 is lower in the two patient lines, particularly line 519 and the distribution is also altered, with less dense TUJ1 staining in the outer regions of the patient organoids relative to the WT (Figure 2B). However, this phenotype is not observed in the KO line. Normalization to the WT organoids shows that the NGLY1 519 and 594 organoids at 45 days expressed around seven- and 1.5-fold less of the TUJ1 levels, respectively (Supplementary Figure S1). Interestingly, this effect was not observed for the NGLY1 KO line until the 75-day time point (Figure 2B). Overall, TUJ1 staining is significantly reduced in patient lines specifically at the 45-day timepoint. This may indicate that the lack of NGLY1 causes neuronal progenitor cells to develop into mature neurons at a slower rate than those in the WT line.

CAS staining by IF and IHC is diffuse in each line at day 7, when EBs develop, but progressively localizes to the center of the hMOs in each line as the organoids mature and grow (Figures 2A,C). Interestingly, quantification of the brightfield CAS-positive regions shows that for the first month, the WT line has around 4-fold more cleaved CAS than that of the NGLY1 519 and NGLY1 594 lines and around 2-fold more cleaved CAS detected than that of the NGLY1 KO line (Figure 2D; Supplementary Figure S1B). As with the TUJ1 staining, this difference diminishes over time, except for the NGLY1 594 line which retains a 4-fold difference in up to 3 months. Additionally, while the staining pattern of most CAS-positive cells shows clear process-like structures in the WT, NGLY1 519 and NGLY1 KO lines, this pattern is absent in the NGLY1 594 line (Figure 2C). It has been observed that CAS activation plays an important role in neuron pruning and programmed cell death, that plays a critical role in the proper remodeling and synaptic development of the early brain (D'Amelio et al., 2010). Based on this observation, the reduced CAS cleavage in the NGLY1 deficient patient lines at early time points (day 7 through day 45) might contribute to the slower development of these midbrain organoids.

### NGLY1 patient-derived midbrain organoids show reduced dopaminergic neuron formation

**FIGURE 3:**
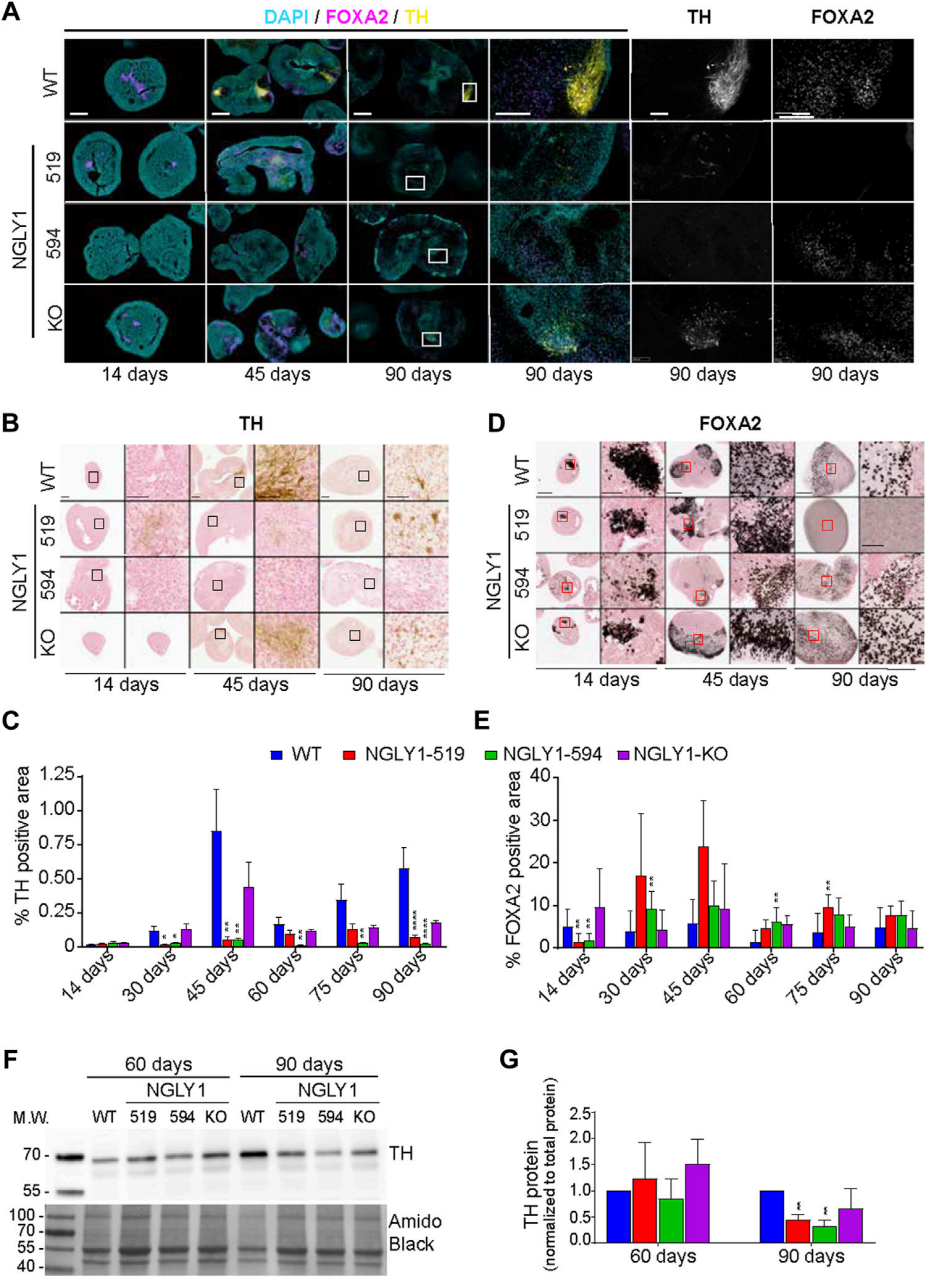
Expression of neuronal markers tyrosine hydroxylase (TH) and FOXA2 in midbrain organoids. **(A)** Representative immunofluorescence images at ×5 and ×20 magnification showing DAPI (cyan), FOXA2 (magenta) and TH (yellow). Scale bars: 250um (5x) and 50um (20x), white boxes indicate zoomed in region. **(B)** Representative bright field images of TH immunohistochemistry staining at ×4 and ×20 magnification. Scale bars: 250um (4x) and 50um (20x), black boxes indicate zoomed in region. **(C)** Quantification of total percent positive TH staining. **(D)** Representative bright field images of FOXA2 immunohistochemistry staining at ×4 and ×20 magnification. Scale bars: 250um (4x) and 50um (20x), red boxes indicate zoomed in region. **(E)** Quantification of total percent positive FOXA2 staining. **(F)** A representative western blot showing TH expression in cell lysates obtained from control and NGLY1 disease midbrain organoids. **(G)** Quantification of TH protein expression normalized to total protein. Data representative of all slices derived from three midbrain organoids. Statistical significance determined by multiple *t*-test analysis denoted by * = (*p* ≤ 0.05), ** = (*p* ≤ 0.01), *** = (*p* ≤ 0.001), **** = (*p* ≤ 0.0001).

Midbrain dopaminergic neurons have a variety of important functions including the control of body movements (Jo et al., 2016). As neuromotor impairment is a common NGLY1 phenotype, we sought to examine dopaminergic neuron formation in NGLY1 deficient midbrain organoids. Forkhead box protein A2 (FOXA2) is a transcription factor necessary for dopaminergic neuron differentiation (Arenas, 2008; Domanskyi et al., 2014; Kim et al., 2019). Loss of FOXA2 results in the reduction of tyrosine hydroxylase (TH) positive dopaminergic neurons (Domanskyi et al., 2014; Kim et al., 2017). TH is a key enzyme in the catecholaminergic pathway and is a marker for dopaminergic neurons. To quantify dopaminergic neuron formation in NGLY1 midbrain organoids we performed immunohistochemical and immunofluorescent staining of dopaminergic neuron markers FOXA2 and TH (Figures 3A,B,D; Supplementary Figure S1C, D).

The results showed that NGLY1 deficient midbrain organoids have significantly lower levels of TH positive neurons. At early time points (day 14, day 30, and day 45), no TH positive neurons were observed in the NGLY1 519 and NGLY1 594 lines, while the NGLY1-KO line developed a similar abundance of TH neurons as the WT organoids (Figures 3B,C). TH expression reached maximum at day 45 and accounted for 1% of organoid slice area. At day 30 and day 45, the difference in TH positive neurons between NGLY1 519, NGLY1 594 and WT was significant. By days 60 and 75 only NGLY1 594 midbrain organoids showed a significant reduction in TH positive neurons, however, by day 90, all three NGLY1 deficient lines showed significant reduction in TH positive neurons compared to the WT line. This observation is corroborated by western blot analysis showing a significant reduction in TH protein levels in the NGLY1 519 and NGLY1 594 lines at day 90 (Figures 3F,G). Although TH neuron count is similar to WT for NGLY1-519 and NGLY1-KO at intermediate time points, their localization differs from WT midbrain organoids. In the WT midbrain organoids, the TH positive neurons group together at the outer edges of the organoid. In the diseased and NGLY1 knockout midbrain organoid, the TH positive neurons sparsely spread throughout the midbrain organoid without forming any clusters.

FOXA2 expression followed the opposite trend compared to TH expression for most time points (Figures 3D,E). Although at day 14, FOXA2 expression is greater in the WT midbrain organoids than in NGLY1 disease model organoids, at all the latter time points, the NGLY1 deficient lines exhibit greater FOXA2 expression than the WT line. At day 45, FOXA2 positive staining in the NGLY1 519 line is roughly 6-fold higher in than WT, while in the NGLY1 594 line is 2.2-fold greater than WT. This trend continues to peak up to day 60, where all three NGLY1 deficient lines show four- to six-fold higher FOXA2 expression compared to the WT line. At the final time points of days 75 and 90, FOXA2 expression was similar to WT for all lines.

Overall, TH expression is significantly reduced in patient lines at most timepoints. Our results suggest that dopaminergic neuronal development is delayed in NGLY1 deficient midbrains and that dopaminergic neurons are not localized to specific regions of the midbrain as they are in healthy midbrains. Together, these results suggest that neuromotor impairment and development delay observed in NGLY1 deficient patients may be attributed to altered dopaminergic neuron formation and localization.

### Astrocyte development is altered in NGLY1 deficient midbrain organoids

**FIGURE 4:**
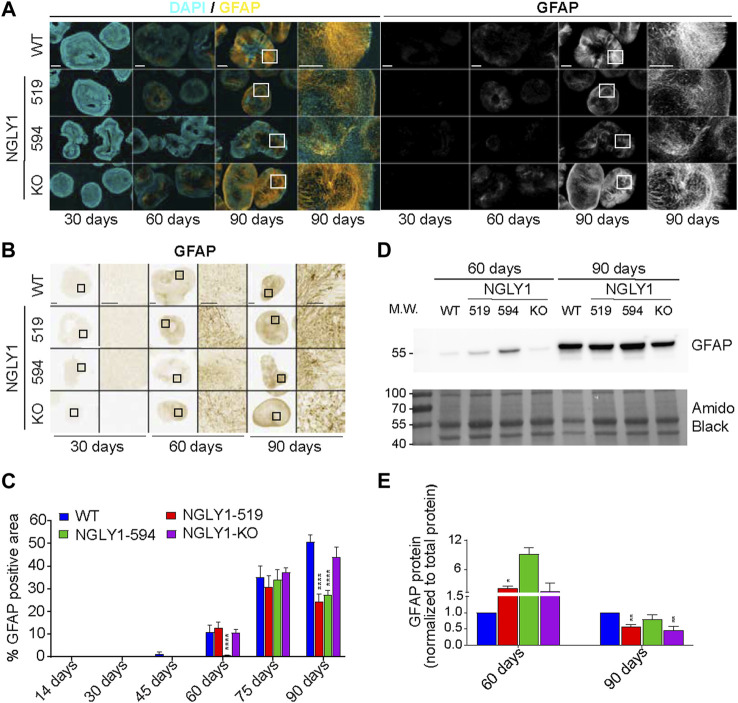
Development of astrocytes in midbrain organoids characterized by GFAP expression. **(A)** Representative immunofluorescence images at ×5 and ×20 magnification showing DAPI (cyan) and GFAP (yellow). Scale bars: 250um (5x) and 50um (20x), white boxes indicate zoomed in region. **(B)** Representative bright field images of GFAP immunohistochemistry staining at ×4 and ×20 magnification. Scale bars: 250um (4x) and 50um (20x), black boxes indicate zoomed in region. **(C)** Quantification of total percent positive GFAP staining. **(D)** A representative western blot showing GFAP expression in cell lysates obtained from control and NGLY1 disease midbrain organoids. **(E)** Quantification of GFAP protein expression normalized to total protein. Data representative of all slices derived from three midbrain organoids. Statistical significance determined by multiple *t*-test analysis denoted by * = (*p* ≤ 0.05), ** = (*p* ≤ 0.01), *** = (*p* ≤ 0.001), **** = (*p* ≤ 0.0001).

GFAP (glial fibrillary acid protein) is an intermediate filament protein that is the established marker for astrocytes (Kawada et al., 2014; Tieng et al., 2014; Zafeiriou et al., 2020). GFAP mutations and altered expression are associated with CNS defects. Patients with altered GFAP expression exhibit an array of disease phenotypes that include macrocephaly, seizures, and psychomotor defects (Paulsen et al., 2022). The clinical phenotypes of altered GFAP expression are similar to those observed in NGLY1 deficient patients (Lam et al., 2017). To evaluate the role of GFAP expression in NGLY1 midbrain organoids we performed immunofluorescent and immunohistochemical staining of GFAP (Figures 4A,B). In WT organoids, GFAP expression was first observed at day 45 of organoids and continued to rise to day 90. At intermediate time points (day 45 and day 75) GFAP expression was variable in the NGLY1 deficient organoids (Figure 4C; Supplementary Figure S1E), with expression significantly lowered in the NGLY1-594 midbrain organoids at day 60. Importantly, at 90 days, GFAP expression was significantly lowered in both patient derived midbrain organoids (51% lower in NGLY1-519% and 36% in NGLY1-594) (Figure 4C; Supplementary Figure S1E). No significant difference was observed in NGLY1-KO midbrain organoids compared to that in WT organoids. Western blot analysis confirmed reduced GFAP protein levels in the 90-day old NGLY1 deficient midbrain organoids (Figure D-E). Discrepancies between immunofluorescence staining and western blot data at the day 60 time point could be attributed to a combination of either different primary antibodies used for the two applications or variable background staining in the NGLY1-594 organoids. Overall, these results suggest that GFAP expression is altered in NGLY1 deficient midbrain development and may contribute to psychomotor defects and seizures observed in NGLY1 deficient patients.

**FIGURE 5:**
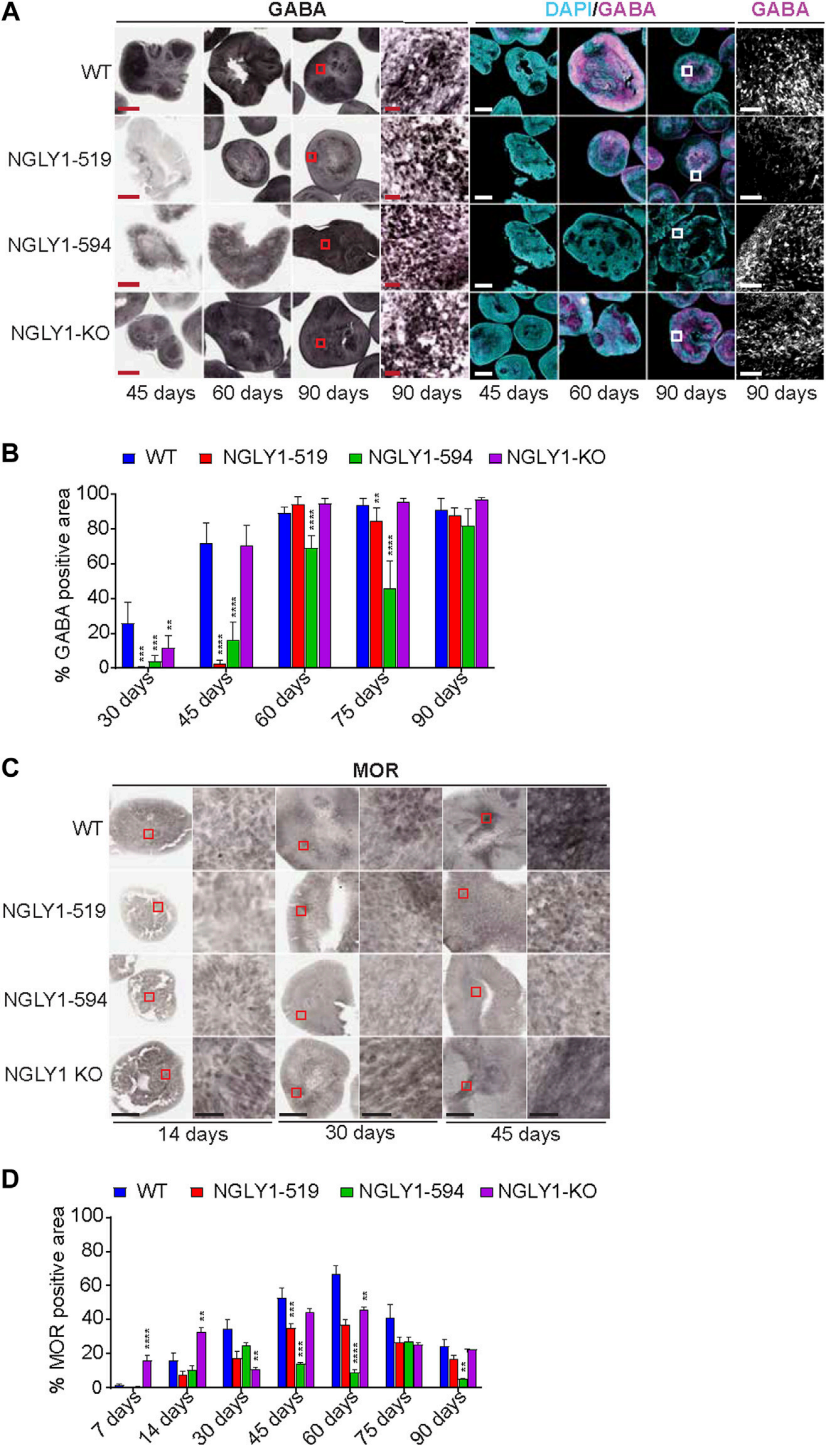
Expression of GABA and MOR in NGLY1 midbrains. **(A)** Representative images using brightfield and immunofluorescence microscopy for WT, NGLY1 diseased and NGLY1 KO midbrain organoids stained with antibody against GABA at different timepoints (45, 60 and 90 days). Scale bars: 500 µM (for 45, 60 and 90 days) and 50 µM (for 90 days zoomed in image). **(B)** Graph showing percent total GABA positive signal in different organoids at different timepoints. **(C)** Representative brightfield immunohistochemistry images for MOR expression at ×4 and ×40 magnification. Scale bars: 250um (4x) and 25um (40x), red boxes indicate zoomed in region. **(D)** Quantification of total percent positive MOR staining. Data representative of all slices derived from three midbrain organoids. Statistical significance determined by multiple *t*-test analysis denoted by * = (*p* ≤ 0.05), ** = (*p* ≤ 0.01), *** = (*p* ≤ 0.001), **** = (*p* ≤ 0.0001).

GABA production is slower in NGLY1 deficient midbrain organoids. GABAergic neurons are significantly expressed in the midbrain region and are known to be involved in regulating the dopaminergic system (Jo et al., 2016; Bouarab et al., 2019). We assessed the production of GABA by direct immunohistochemical staining for GABA at different time points (Figures 5A,B; Supplementary Figure S1F). WT midbrain organoids showed significant GABA staining (about 20% of analyzed organoid area) in 30-day old organoids. By day 45 and beyond, GABA neurotransmitter could be detected in 80%–90% of the WT organoids. Interestingly, both NGLY1-519 and NGLY1-594 midbrain organoids showed a 70%–90% reduction in GABA at the day 30 and day 45-day time points compared to WT midbrain organoids. Both the patient lines however caught up with WT organoids in terms of GABA production at day 90, suggesting a delay rather than inability to develop GABAergic neurons or GABA related pathways in NGLY1 deficient organoids. The NGLY1 KO organoids showed about 50% lower GABA staining compared to WT organoids only at the day 30 time point and were able to catch up with the WT organoids faster compared to the NGLY1-519 and NGLY1-594 lines. Overall, the NGLY1 patient lines showed a reduced production of GABA neurotransmitter at the 30 and 45-day timepoints, suggesting that GABAergic pathway development is delayed during midbrain development in NGLY1 disease organoids.

### Disease and KO NGLY1 midbrain organoids fail to develop robust MOR expression

The diminished pain sensation was reported in patients NGLY1 deficiency along with severe neuropathy (Caglayan et al., 2015). The mu opioid receptor (MOR), a G protein-coupled receptor, is a part of the brain’s pain management system and an important regulator of dopaminergic and GABAergic neurons found in the midbrain (Bouarab et al., 2019). Based on this information, we assessed MOR expression in the midbrain organoids (Figure 5C). In WT organoids, the MOR expression increased from day 14 to day 60 and thereafter decreased consistently (Figure 5D). The NGLY1 deficient lines did not follow this same trend, and normalization to WT showed that the WT line consistently expressed 1.5 to 4-fold more MOR than the NGLY1-519 and NGLY1 KO lines, and around 8-fold more MOR than the NGLY1-594 line over the entire observed period (Figure 5D; Supplementary Figure S1G). Additionally, relative to the patient-derived and KO lines, MOR expression in the WT line is much more localized to specific edge regions of the organoids (Figure 5C), similar to the TH localization (Figure 3B).

**FIGURE 6:**
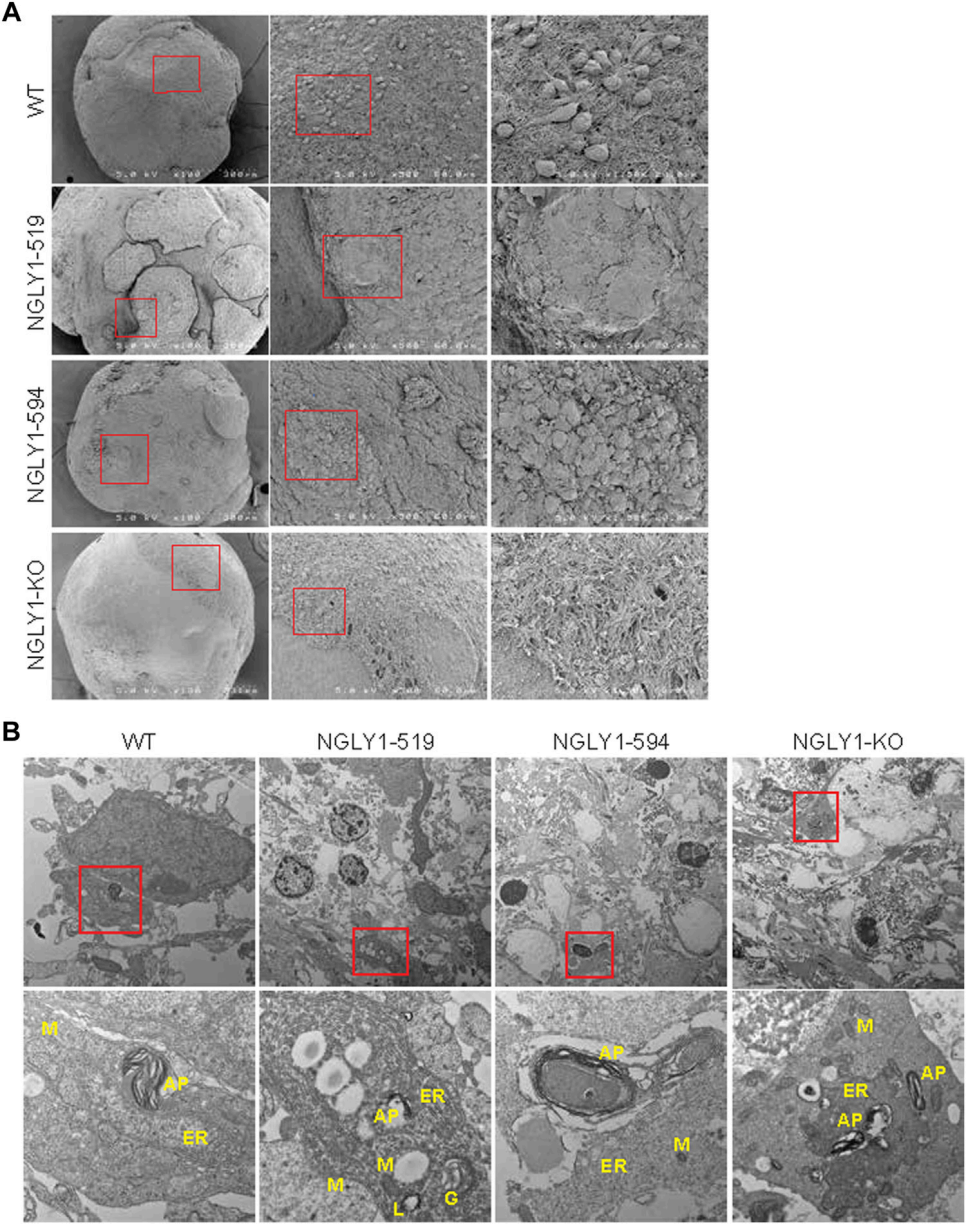
Electron microscopy characterization of midbrain organoids. **(A)** SEM images at 100x, ×500 and ×1500 magnification. **(B)** TEM images at 1000x and 6000x magnification showing cellular organelles (ER-endoplasmic reticulum, M-mitochondria, L-lysosome, G-golgi, AP-autophagosome).

Electron microscopy reveals structural differences in NGLY1 deficient midbrain organoids We performed scanning electron microscopy (SEM) and transmission electron microscopy (TEM) to analyze the overall topography of the midbrain organoids and the subcellular structures of the cells within midbrain organoids (Figure 6). Compared to the WT organoids, the NGLY1 diseased midbrain organoids appeared smoother and with less obvious cell bodies, although the NGLY1 KO line showed this effect to a lower degree (Figure 6A). This observation concurs with our previous result showing reduced TUJ1 staining on the outer region of the NGLY1 deficient organoids. This phenomenon could be partly attributed to reduced expression of extracellular matrix proteins in NGLY1 deficient organoids, as determined by proteomic and RNA-seq analysis in this study. By TEM analysis regions with accumulation of vesicles, including autophagosomes, were more often found in NGLY1 deficient lines. Additionally, autophagosomes in the NGLY1 patient lines appeared to be larger in size (Figure 6B). This observation is in line with recent findings indicating dysregulated autophagy associated with NGLY1 deficiency (Mueller et al., 2020; Needs et al., 2022).

### Global genomic and proteomic expression profiling of NGLY1 midbrain organoids

**FIGURE 7:**
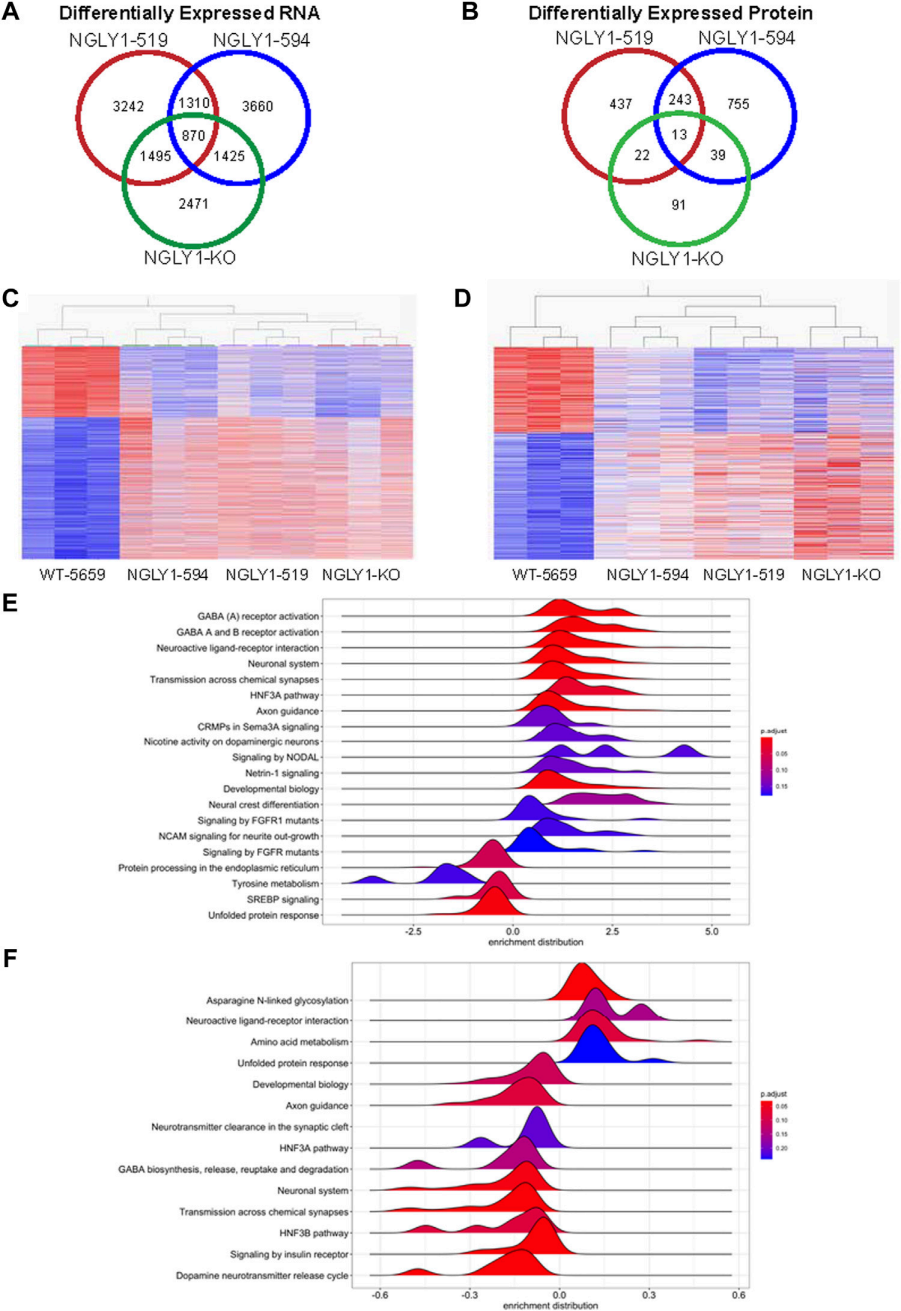
Multiomic analysis of NGLY1 deficient midbrain organoids. **(A)** Venn diagram summary of differentially expressed genes in midbrain organoids, differential expression was defined as absolute value log-2 fold-change >1 with a FDR <0.01. **(B)** Venn diagram summary of differentially expressed proteins in midbrain organoids, differential expression was defined as absolute value fold-change > |25%| with a FDR <0.05. **(C)** Heatmap showing upregulated and downregulated transcripts in NGLY1 deficient midbrain organoids. Expression normalized to wild type, red corresponds to higher expression, blue represents lower expression. **(D)** Heatmap showing upregulated and downregulated proteins in NGLY1 deficient midbrain organoids. Expression normalized to wild type, red corresponds to higher expression, and blue represents lowered expression. **(E)** Gene set enrichment analysis of global transcriptomic analysis of NGLY1 deficient midbrain organoids using the NCATS BioPlanet pathway annotation, pathways with an FDR < 0.20 were included. **(F)** Gene set enrichment analysis of proteomic profiling of NGLY1 deficient midbrain organoids utilizing the NCATS BioPlanet pathway database, pathways with an FDR < 0.25 were included.

RNA-seq analysis and quantitative proteomic profiling was carried out on NGLY1 midbrain organoids at day 90. Genes were determined to be differentially expressed with a cutoff of a Log 2-Fold Change greater than |1| and a FDR below 1%, proteins were determined to be differentially expressed with a 30% change and an FDR below 5%. NGLY1-519 and NGLY1-594 harbor different NGLY1 mutations and share 1310 genes and 243 proteins that are similarly differentially expressed (Figures 7A, B). The CRISPR generated NGLY1 knockout exhibited a different genomic and proteomic profile than the patient derived midbrain organoids. Including the data from the differential expression analysis of the NGLY1-KO midbrain organoids; there were 870 genes differentially expressed and 13 proteins.

We performed ranked gene set enrichment analysis (GSEA) on patient derived NGLY1 deficient midbrain organoids on the genomic and proteomic data using the NCATS BioPlanet database for pathway classification (Figures 7E, F). GSEA analysis indicated a significant upregulation of gene sets associated with GABA receptor activation, neuronal system function, transmission across chemical synapses and the HNF3A pathway. Interesting downregulated pathways include protein processing in the ER and tyrosine metabolism.

GSEA analyses of proteomic data were consistent with enriched pathways identified by RNA-seq results but differed in direction of enrichment (Figure 7F). Proteomic analysis revealed that asparagine N-linked glycosylation, amino acid metabolism pathways were upregulated. Conversely GABA biosynthesis and related pathways were found to be downregulated, opposite of the genomic analysis. Additionally, proteomic analysis indicated that proteins associated with dopamine transmitter release, transmission across chemical synapses, the HNF3B pathway, and pathways associated with neuronal function were also identified as significantly enriched.

## Discussion

NGLY1 deficiency is an ultrarare genetic disorder that exhibits a complex clinical symptom that include seizures, hyperkinetic movement, alacrima, developmental delay, and abnormal liver function. While the patient population is small, the disease phenotype of NGLY1 deficiency may help to characterize the biological functions of NGLY1 that were unknown before NGLY1 deficiency was reported. To better model NGLY1 deficiency and study disease pathophysiology, we generated midbrain organoids from NGLY1 patient-derived iPSCs and studied developmental changes of several markers at different time points up to 90 days.

The WT organoids reached their peak size as well as peak expression for most tested markers around the 45–60-day time period. Two (NGLY1 519 and NGLY1 KO lines) out of the three NGLY1 deficient lines produced midbrain organoids that were smaller in size and showed slower midbrain development compared to WT organoids. Total neuronal marker TUJ1 and astrocytic marker GFAP were both reduced at different development time points during the NGLY1 deficient organoid development. This could be attributed to either reduced differentiation potential in NGLY1 deficient lines or loss of neuronal cells during development. This phenotype aligns well with the reports that mutations in *NGLY1* linked to the general delay in brain development observed in patients. The SEM imaging at day 90 reveals that there remains a qualitative difference in the density of the ECM between the mutant and WT lines. Additionally, we noted a buildup in autophagosomes in the NGLY1 organoids, which further support the dysregulation of autophagy in NGLY1 deficiency (Mueller et al., 2020), though it remains unclear if the buildup of autophagosomes is due to autophagy activation or late-stage inhibition (Yoshii and Mizushima, 2017) that will need to be further investigated.

Differences were observed in the staining of MOR and GFAP, which adds additional clinical relevance to the model as insensitivity to pain has been noted in patients along with neurodegenerative symptoms associated with dysregulated astrocytes (Caglayan et al., 2015; Asahina et al., 2020; Mueller et al., 2020; Needs et al., 2022). In most cases the midbrain organoids from the NGLY1 KO line presented a phenotype between the WT and NGLY1 patient organoids for the previously mentioned markers. This may suggest that genetic variations in the patient-derived organoids can also contribute to disease phenotypes. Additionally, we could not rule out the possibility of clonal variations, but this is an inherent issue in most iPSC studies. Another possibility is the differences of iPS cell generation as the NGLY1 iPS lines were generated from the NGLY1 fibroblasts derived from the patients and the NGLY1 KO iPS cell line was generated in WT iPS cells by CRISPR. Therefore, these observed differences in NGLY1 patient organoids and NGLY1 KO organoids will need further study.

Multiomic analysis of NGLY1 midbrain organoids is challenging due to the cell-type heterogeneity of brain organoids. However, our results provide insights into the molecular pathology of NGLY1 deficiency. Consistent with other studies, we observed an upregulation of the unfolded protein response (UPR) in patient derived NGLY1 deficient brain organoids using global proteomic profiling (Owings et al., 2018; Mueller et al., 2020; Talsness et al., 2020). However, we did not observe a significant upregulation of UPR gene expression. Additionally, NGLY1-KO brain organoids exhibit a distinctly different molecular phenotype by RNA-seq and proteomics compared to NGLY1 deficient patient derived brain organoids. This phenomenon is consistent in patient derived neural stem cells and patient derived neuronal models (data not shown) showing the importance of using patient derived *in vitro* systems to better understand the pathology of NGLY1 deficiency and other diseases.

The NGLY1 patient derived midbrain organoids showed almost an 80%–95% reduction in dopaminergic neuronal marker tyrosine hydroxylase (TH), suggesting a possible loss or malformation of dopaminergic neurons in NGLY1 disorder. This finding is also supported by differences in FOXA2 expression at different time points of NGLY1 deficient midbrain organoid development. The reduced FOXA2 expression in the early stages of NGLY1 deficient midbrain organoid development could suggest reduced specification of dopaminergic neurons, whereas the increased FOXA2 expression in the mature NGLY1 deficient organoids could possibly be a compensatory mechanism to the loss of dopaminergic neurons (Smits et al., 2019). Interestingly, preliminary data with western blot analysis did not show a corresponding reduction of other dopaminergic markers like vesicular monoamine transporter 2 (VMAT2) and dopamine transporter (DAT) (Supplementary Figure S2) in NGLY1 deficient organoids. This could hint at merely a reduction in TH expression and not loss of dopaminergic neurons. More in-depth study in a dopaminergic neuronal model of NGLY1 disease is required to understand the disruption of the dopaminergic system in NGLY1 disorder. Non-etheless, our results are consistent with findings from two drug repurposing screens in worm (Iyer et al., 2019) and fly models of NGLY1 deficiency (Hope et al., 2022), which identified compounds that modulate the dopamine pathway to reverse NGLY1 related phenotype. Additionally, the NGLY1 deficient organoids showed a reduction in GABA neurotransmitter up to day 45. Interestingly, GABA neurons are widely expressed in the ventral tegmental area (VTA) of the midbrain and are known to form synaptic connections with and inhibit dopaminergic neurons in the area (Bouarab et al., 2019). The reduction in GABA neurotransmitter in NGLY1 deficient organoids could be a feedback mechanism in response to the reduced dopaminergic tone in these organoids. Taken together, the trends in expression of TH and GABA neurotransmitter suggests a possible disruption of upstream signaling pathways, specifically in the midbrain VTA. This is an important finding since patients with NGLY1 deficiency display symptoms related to movement disorder.

## Data Availability

The data presented in the study are deposited in the Gene Expression Omnibus repository, accession number GSE224294, https://www.ncbi.nlm.nih.gov/geo/query/acc.cgi?acc=GSE224294.

## Supplementary material

Supplementary Figure S1 Expression of different markers in NGLY1 deficient midbrain organoids relative to WT. Quantification of **(A)** TUJ1, **(B)** caspase 3 (CAS), **(C)** tyrosine hydroxylase (TH), **(D)** FOXA2, **(E)** GFAP, **(F)** GABA and **(G)** MOR staining shown as relative fold change compared to wild type midbrain organoid at different time points. Data representative of all slices derived from three midbrain organoids. Statistical significance determined by multiple t-test analysis denoted by * = (*p* ≤ 0.05), ** = (*p* ≤ 0.01), *** = (*p* ≤ 0.001), **** = (*p* ≤ 0.0001).

Supplementary Figure S2 Expression of dopaminergic neuron markers in NGLY1 deficient midbrain organoids. Representative western blot showing **(A)** vesicular monoamine transporter 2 (VMAT2) and **(B)** dopamine transporter (DAT) protein expression normalized to total protein.

## References

[B1] Arenas E. (2008). Foxa2: The rise and fall of dopamine neurons. Cell Stem Cell 2, 110–112. 10.1016/j.stem.2008.01.012 18371430

[B2] Asahina M. Fujinawa R. Nakamura S. Yokoyama K. Tozawa R. Suzuki T. (2020). Ngly1 -/- rats develop neurodegenerative phenotypes and pathological abnormalities in their peripheral and central nervous systems. Hum. Mol. Genet. 29, 1635–1647. 10.1093/hmg/ddaa059 32259258 PMC7322575

[B3] Bouarab C. Thompson B. Polter A. M. (2019). VTA GABA neurons at the interface of stress and reward. Front. Neural Circuits 13, 78. 10.3389/fncir.2019.00078 31866835 PMC6906177

[B4] Caglayan A. O. Comu S. Baranoski J. F. Parman Y. Kaymakçalan H. Akgumus G. T. (2015). NGLY1 mutation causes neuromotor impairment, intellectual disability, and neuropathy. Eur. J. Med. Genet. 58, 39–43. 10.1016/j.ejmg.2014.08.008 25220016 PMC4804755

[B5] D'Amelio M. Cavallucci V. Cecconi F. (2010). Neuronal caspase-3 signaling: Not only cell death. Cell Death Differ. 17, 1104–1114. 10.1038/cdd.2009.180 19960023

[B6] Domanskyi A. Alter H. Vogt M. A. Gass P. Vinnikov I. A. (2014). Transcription factors Foxa1 and Foxa2 are required for adult dopamine neurons maintenance. Front. Cell Neurosci. 8, 275. 10.3389/fncel.2014.00275 25249938 PMC4158790

[B7] Han S. Y. Pandey A. Moore T. Galeone A. Duraine L. Cowan T. M. (2020). A conserved role for AMP-activated protein kinase in NGLY1 deficiency. PLoS Genet. 16, e1009258. 10.1371/journal.pgen.1009258 33315951 PMC7769621

[B8] Hope K. A. Berman A. R. Peterson R. T. Chow C. Y. (2022). An *in vivo* drug repurposing screen and transcriptional analyses reveals the serotonin pathway and GSK3 as major therapeutic targets for NGLY1 deficiency. PLoS Genet. 18, e1010228. 10.1371/journal.pgen.1010228 35653343 PMC9162339

[B9] Iyer S. Mast J. D. Tsang H. Rodriguez T. P. DiPrimio N. Prangley M. (2019). Drug screens of NGLY1 deficiency in worm and fly models reveal catecholamine, NRF2 and anti-inflammatory-pathway activation as potential clinical approaches. Dis. Model Mech. 12, dmm040576. 10.1242/dmm.040576 31615832 PMC6899034

[B10] Jo J. Xiao Y. Sun A. X. Cukuroglu E. Tran H. D. Göke J. (2016). Midbrain-like organoids from human pluripotent stem cells contain functional dopaminergic and neuromelanin-producing neurons. Cell Stem Cell 19, 248–257. 10.1016/j.stem.2016.07.005 27476966 PMC5510242

[B11] Kawada K. Iekumo T. Saito R. Kaneko M. Mimori S. Nomura Y. (2014). Aberrant neuronal differentiation and inhibition of dendrite outgrowth resulting from endoplasmic reticulum stress. J. Neurosci. Res. 92, 1122–1133. 10.1002/jnr.23389 24723324 PMC4320781

[B12] Kim H. Park H. J. Choi H. Chang Y. Park H. Shin J. (2019). Modeling G2019S-LRRK2 sporadic Parkinson's disease in 3D midbrain organoids. Stem Cell Rep. 12, 518–531. 10.1016/j.stemcr.2019.01.020 PMC6410341 30799274

[B13] Kim T. Song J. J. Puspita L. Valiulahi P. Shim J. W. Lee S. H. (2017). *In vitro* generation of mature midbrain-type dopamine neurons by adjusting exogenous Nurr1 and Foxa2 expressions to their physiologic patterns. Exp. Mol. Med. 49, e300. 10.1038/emm.2016.163 28280264 PMC5382556

[B14] Lam C. Ferreira C. Krasnewich D. Toro C. Latham L. Zein W. M. (2017). Prospective phenotyping of NGLY1-CDDG, the first congenital disorder of deglycosylation. Genet. Med. 19, 160–168. 10.1038/gim.2016.75 27388694 PMC7477955

[B15] Li R. Pradhan M. Xu M. Baskfield A. Farkhondeh A. Cheng Y. S. (2019). Generation of an induced pluripotent stem cell line (TRNDi002-B) from a patient carrying compound heterozygous p.Q208X and p.G310G mutations in the NGLY1 gene. Stem Cell Res. 34, 101362. 10.1016/j.scr.2018.101362 30612078 PMC6492929

[B16] Monzel A. S. Smits L. M. Hemmer K. Hachi S. Moreno E. L. van Wuellen T. (2017). Derivation of human midbrain-specific organoids from neuroepithelial stem cells. Stem Cell Rep. 8, 1144–1154. 10.1016/j.stemcr.2017.03.010 PMC5425618 28416282

[B17] Mueller W. F. Jakob P. Sun H. Clauder-Münster S. Ghidelli-Disse S. Ordonez D. (2020). Loss of N-glycanase 1 alters transcriptional and translational regulation in K562 cell lines. G3 (Bethesda, Md.) 10, 1585–1597. 10.1534/g3.119.401031 32265286 PMC7202010

[B18] Needs S. H. Bootman M. D. Grotzke J. E. Kramer H. B. Allman S. A. (2022). Off-target inhibition of NGLY1 by the polycaspase inhibitor Z-VAD-fmk induces cellular autophagy. FEBS J. 289, 3115–3131. 10.1111/febs.16345 34995415 PMC9304259

[B19] Owings K. G. Lowry J. B. Bi Y. Might M. Chow C. Y. (2018). Transcriptome and functional analysis in a Drosophila model of NGLY1 deficiency provides insight into therapeutic approaches. Hum. Mol. Genet. 27, 1055–1066. 10.1093/hmg/ddy026 29346549 PMC5886220

[B20] Paulsen B. Velasco S. Kedaigle A. J. Pigoni M. Quadrato G. Deo A. J. (2022). Autism genes converge on asynchronous development of shared neuron classes. Nature 602, 268–273. 10.1038/s41586-021-04358-6 35110736 PMC8852827

[B21] Qian X. Jacob F. Song M. M. Nguyen H. N. Song H. Ming G. L. (2018). Generation of human brain region–specific organoids using a miniaturized spinning bioreactor. Nat. Protoc. 13, 565–580. 10.1038/nprot.2017.152 29470464 PMC6241211

[B22] Smits L. M. Reinhardt L. Reinhardt P. Glatza M. Monzel A. S. Stanslowsky N. (2019). Modeling Parkinson's disease in midbrain-like organoids. NPJ Park. Dis. 5, 5. 10.1038/s41531-019-0078-4 PMC6450999 30963107

[B23] Suzuki T. Huang C. Fujihira H. (2016). The cytoplasmic peptide: N-Glycanase (NGLY1) - structure, expression and cellular functions. Gene 577, 1–7. 10.1016/j.gene.2015.11.021 26611529 PMC4691572

[B24] Suzuki T. Huang C. Harada Y. Hosomi A. Masahara-Negishi Y. Seino J. (2015). Endo-β-n-acetylglucosaminidase forms N-GlcNAc protein aggregates during ER-associated degradation in NGLY1-defective cells. Proc. Natl. Acad. Sci. U. S. A. 112, 1398–1403. 10.1073/pnas.1414593112 25605922 PMC4321286

[B25] Suzuki T. Kwofie M. A. Lennarz W. J. (2003). Ngly1, a mouse gene encoding a deglycosylating enzyme implicated in proteasomal degradation: Expression, genomic organization, and chromosomal mapping. Biochem. Biophysical Res. Commun. 304, 326–332. 10.1016/S0006-291X(03)00600-4 12711318

[B26] Suzuki T. (2015). The cytoplasmic peptide:N-glycanase (Ngly1) - basic science encounters a human genetic disorder. J. Biochem. 157, 23–34. 10.1093/jb/mvu068 25398991

[B27] Talsness D. M. Owings K. G. Coelho E. Mercenne G. Pleinis J. M. Partha R. (2020). A Drosophila screen identifies NKCC1 as a modifier of NGLY1 deficiency. Elife 9, e57831. 10.7554/eLife.57831 33315011 PMC7758059

[B28] Tambe M. A. Ng B. G. Freeze H. H. (2019). N-glycanase 1 transcriptionally regulates aquaporins independent of its enzymatic activity. Cell Rep. 29, 4620–4631. 10.1016/j.celrep.2019.11.097 31875565

[B29] Tieng V. Stoppini L. Villy S. Fathi M. Dubois-Dauphin M. Krause K. H. (2014). Engineering of midbrain organoids containing long-lived dopaminergic neurons. Stem Cells Dev. 23, 1535–1547. 10.1089/scd.2013.0442 24576173

[B30] Yoshii S. R. Mizushima N. (2017). Monitoring and measuring autophagy. Int. J. Mol. Sci. 18, 1865. 10.3390/ijms18091865 28846632 PMC5618514

[B31] Zafeiriou M. P. Bao G. Hudson J. Halder R. Blenkle A. Schreiber M. K. (2020). Developmental GABA polarity switch and neuronal plasticity in Bioengineered Neuronal Organoids. Nat. Commun. 11, 3791. 10.1038/s41467-020-17521-w 32728089 PMC7391775

