# Supplementary figures and images for: Generation and characterization of NGLY1 patient-derived midbrain organoids
Source: Front Cell Dev Biol. 2023 Feb 16;11:1039182. doi: 10.3389/fcell.2023.1039182 (PMC9978932; doi:10.3389/fcell.2023.1039182)

**A**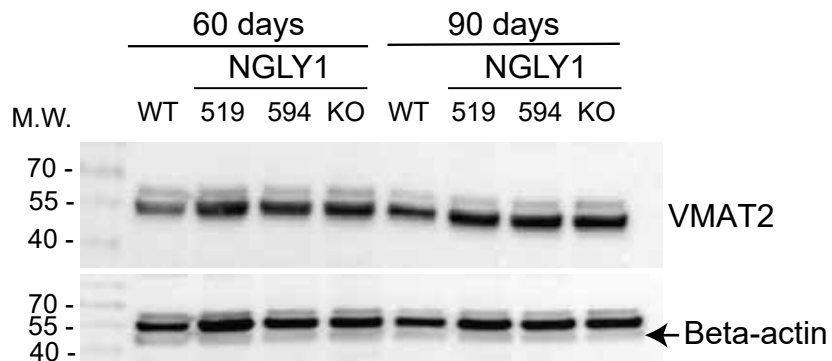**B**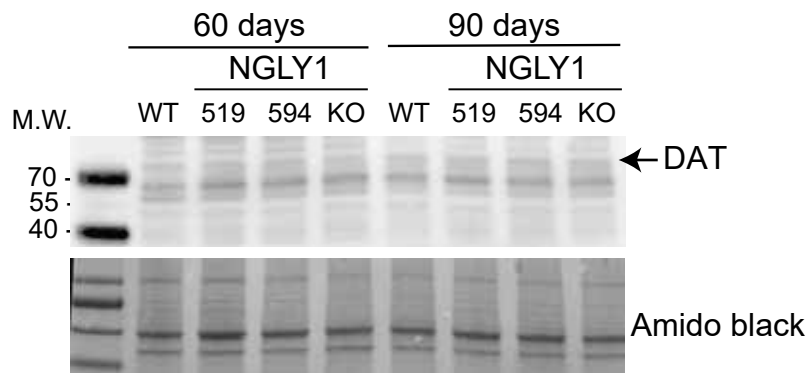

Supplement: Supplementary file 1 [file Image2.pdf]

**A**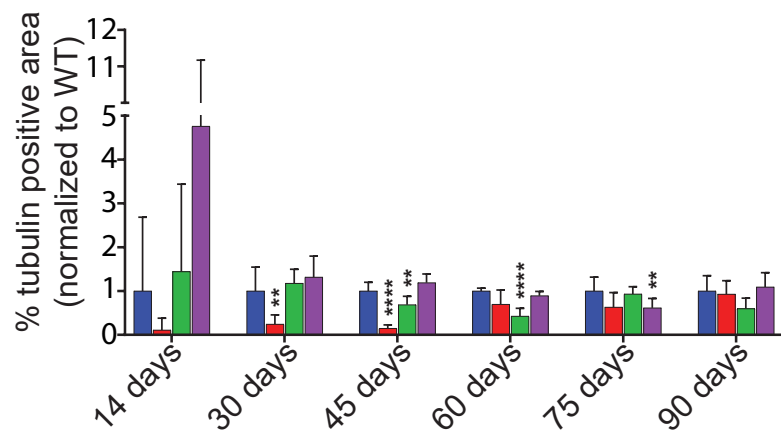**B**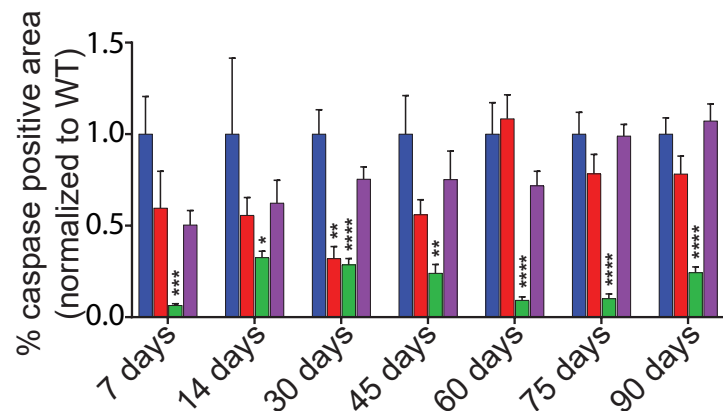**C**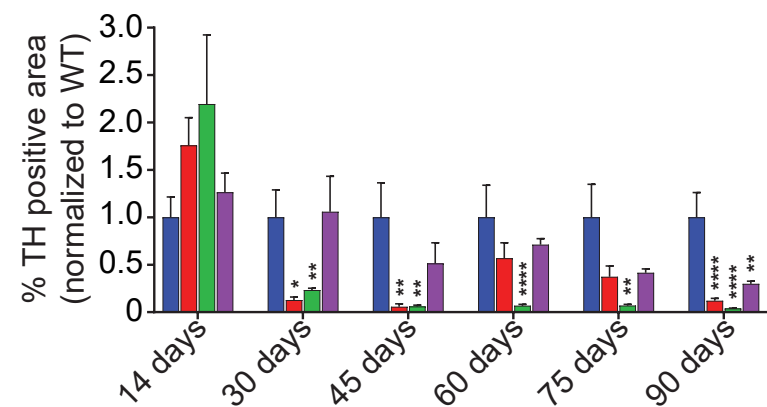**D**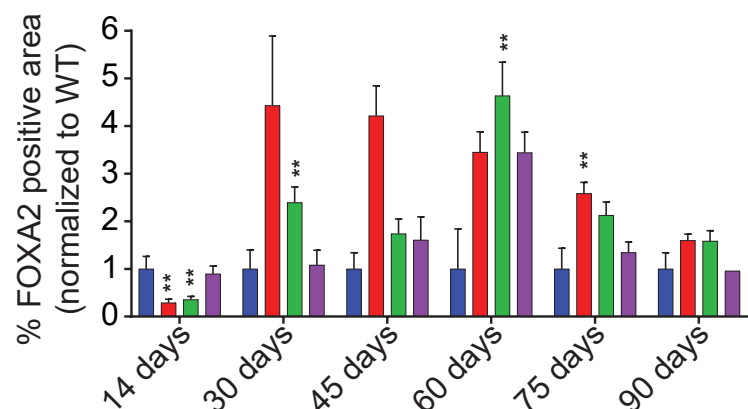**E**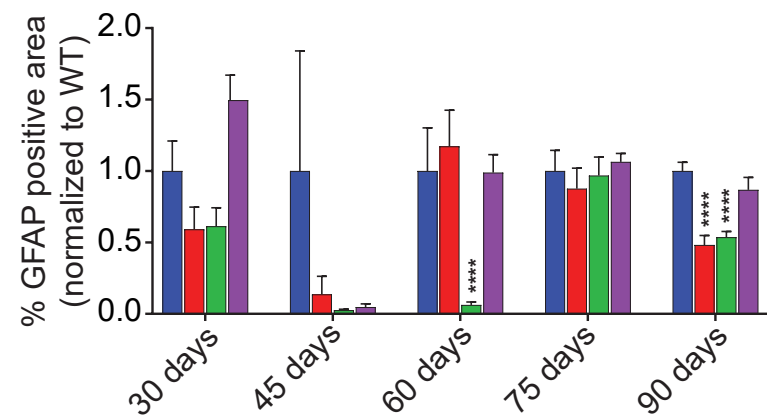**F**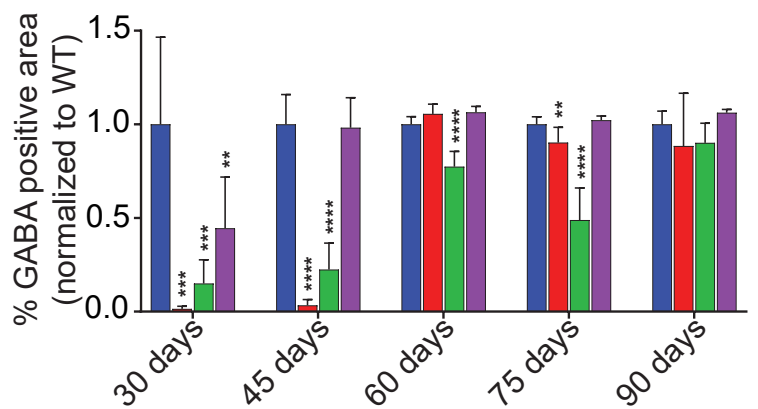**G**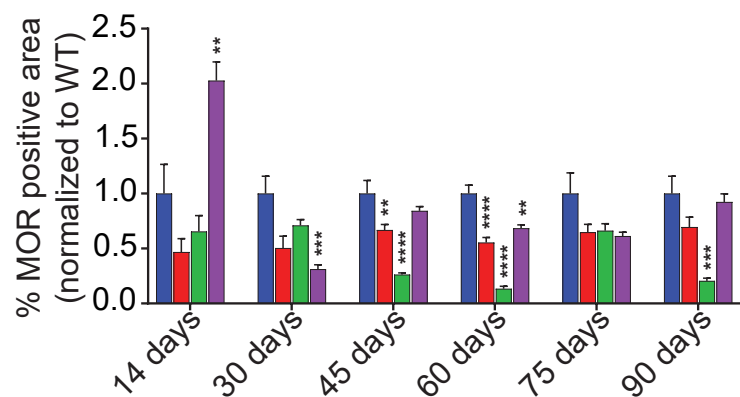

Supplement: Supplementary file 2 [file Image1.pdf]
